# Impact of Biomimetic Fin on Pitching Characteristics of a Hydrofoil

**DOI:** 10.3390/biomimetics10070462

**Published:** 2025-07-15

**Authors:** Faraz Ikram, Muhammad Yamin Younis, Bilal Akbar Chuddher, Usman Latif, Haroon Mushtaq, Kamran Afzal, Muhammad Asif Awan, Asad Ijaz, Noman Bashir

**Affiliations:** 1 Department of Mechanical Engineering, Mirpur University of Sciences and Technology, Mirpur 10250, AJK, Pakistan; faraz.ikram.me@must.edu.pk (F.I.); haroon.me@must.edu.pk (H.M.); kamran.afzal@must.edu.pk (K.A.); asif.me@must.edu.pk (M.A.A.); asadijaz@must.edu.pk (A.I.); noman.me@must.edu.pk (N.B.); 2 Department of Industrial Engineering, King Khalid University, Abha 61421, Saudi Arabia; bmchuddher@kku.edu.sa; 3 Department of Mechanical Engineering and Materials Science, Duke University, Durham, NC 27708-0300, USA; usman.latif@duke.edu

**Keywords:** biomimetic fin, pitching foil, pure/passive pitching, PIV, videography

## Abstract

Biomimetic design for engineering applications may suggest the optimal performance of engineering devices. In this work the passive/pure pitching characteristics of a hydrofoil are investigated experimentally with and without a pair of biomimetic fin strips placed symmetrically on the two sides of the foil leading edge. The work is performed in a recirculating water channel at low Reynolds numbers (Re) with a range of 1300 ≤ Re ≤ 3200. Using high-speed videography and Particle Image Velocimetry (PIV), the pitching characteristics and wakes are visualized. Passive pitching characteristics, i.e., the pitching amplitude and pitching frequency of the hydrofoils, are investigated based on their trailing edge movement. Significant improvement in both pitching frequency and amplitudes are observed for the foil with fin strips compared to the baseline simple foil. Comparing the pitching characteristics of the two foils, it is observed that the hydrofoil with biomimetic fin strips exhibits 25% and 21% higher pitching amplitude and pitching frequency, respectively, compared to that of the baseline at comparable Reynolds numbers. The initiation of pitching for the finned foil is also observed at comparatively low Reynolds numbers. The wake is also studied using time mean and fluctuating velocity profiles obtained using PIV.

## 1. Introduction

The surge in global energy demand, coupled with growing concerns over environmental sustainability, has sparked considerable interest in renewable energy sources in recent years. As a result, there has been a strong focus on the development and utilization of clean, renewable energy alternatives. Consequently, numerous innovative approaches for harnessing energy from oceanic fluid flow are actively being explored [1]. The dynamic response of a structure interacting with fluid flow is referred to as flow-induced vibration (FIV). These induced vibrations can be effectively utilized to generate clean energy using bluff bodies [2]. Oscillating foils are very common in nature. Controlling vortices generated by flapping wings or fins to create propulsion is a common mechanism employed by insects, birds, and aquatic animals. Numerous studies have investigated the dynamics of flapping motion to deepen our understanding of this propulsion mechanism and improve the design of man-made propulsion systems [3,4]. Lower Reynolds numbers reflect flow environments typical of many biological systems, such as the swimming behavior of larval fish (Re ≈ 10–1000), medaka fish (Re ≈ 80–4500), zebrafish (Re ≈ 120–3000), tadpoles (Re ≈ 15–1800), and small aquatic organisms [5,6]. The unsteady yet coherent flow, influenced by both viscous and inertial forces, is optimal for studying vortex-induced motion relevant to bio-inspired systems. Applications include robotic swimmers, microrobots, and energy harvesters operating efficiently in low-to-moderate Reynolds number regimes [7,8,9,10,11,12].

Oscillating airfoils have found extensive application in various engineering systems, including vertical-axis wind turbines, micro-air vehicles, and underwater propulsion systems [13]. One of their key advantages is that they are not subjected to the centrifugal stresses encountered by rotating blades in traditional turbines, which enhances their structural robustness [14]. Moreover, oscillating foils traverse a rectangular flow area that can be relatively wide and shallow thus can be installed in shallow flow, e.g., canals and rivers [15]. Compared to rotary-blade turbines, flapping hydrofoils can achieve greater efficiency at lower flow speeds [9,16]. Moreover, the piezoelectric energy is also harnessed at lower Reynolds numbers [14].

There are three modes of motion of oscillating foil, i.e., plunging (or heaving), pitching, and flapping, which can be fully active, semi-active, and fully passive. These motions have a vital effect on the wake structure and the aero/hydrodynamic performance of oscillating foils [17]. A foil may undergo fully active motion (predefined and controlled) [17,18] or the semi-active or semi-passive motion (one motion is predefined, while the other is driven by the flow) [9]. The performance of both primarily relies on factors such as the location of the pivot point [19], oscillation frequency [20], Reynolds number [21], plunging amplitude [22], pitching amplitude [23,24], the shape of foil [2], aspect ratio, reduced frequency [25], and the angle of attack [26,27,28]. The propulsion efficiency is optimized with different modes of foil flapping [29,30]. While extensive research has been conducted on fully active and semi-active systems, relatively little is focused on fully passive systems [31].

The fully passive mode of energy harvesting [32] provides a mechanical advantage compared to active and semi-active methods, though it loses direct control over the foil’s motion. The heaving and pitching motions are entirely driven by the fluid flow, without actuators to improve the foil’s energy extraction efficiency [33,34]. Qadri et al. [35] utilized a combined pitching and heaving motion to boost the efficiency of the system. Boudreau et al. [36,37] expanded this concept in a simple arrangement, further increasing the efficiency and power coefficient and making it highly relevant for practical applications. Duarte et al. [38] revealed that the pivot axis needs to be positioned beyond 29% of the chord length from the leading edge for the device to function properly. Integrating heaving with a pitching motion significantly improves both the time-averaged thrust and propulsive efficiency [29,39]. Force fluctuations and mechanical complexity in the flapping and heaving motions require coupling the two through intricate mechanisms. This complexity can lead to energy loss from friction, increased failure risk, and higher costs [17,27].

The pitching motion of foil involves the tail of foil moving through up-stroke and down-stroke cycles at a fixed pivot point along the blade’s chord line [13]. Koochesfahani [40] experimentally showed that the pure pitching of an airfoil can generate thrust. He found that thrust occurs above a critical frequency, which decreases as pitch amplitude increases. Godoy Diana et al. [41] identified two key dynamics in the wake vortices of a pitching foil within 100 < Re < 10,000, the transition from drag to thrust. Sarkar and Venkatraman [42] performed numerical simulations at Re ~10,000 and found that oscillation parameters influence the vortex structures and cause wakes to shift from drag to thrust at higher frequencies. Vortex shedding patterns, influenced by body motion and flow conditions, govern the transition from drag- to thrust-generating wakes at different amplitudes and frequencies in flapping foils [43,44,45,46,47].

Forced pitching motion [48] using NACA 0015 airfoil at low Reynolds number concluded that the pitching frequency and amplitudes strongly influence the thrust, power, and efficiency [49]. Zaka et al. [50] improved thrust and efficiency using asymmetrically pitching foil compared to sinusoidal motion due to resonance with shear-layer roll-up. The energy conversion efficiency of passive pitching motions of a hydrofoil was observed to be 0.13 [51]. Mackowski et al. [52] observed 12% propulsive efficiency of a NACA 0012 airfoil in pure pitching motion at a low Reynolds number, which was relatively low.

The efficient propulsion mechanisms are achieved by the distinct shapes, sizes, and movements of fish fins and tails [53]. Bio-inspired modifications to airfoils and wings have been extensively studied due to their potential for enhancing performance and adaptability. By changing the shape of the bluff body, it can cause turbulence and vorticity as a result of splitting the incoming flow [54,55]. The leading edge vortices (LEVs) play a pivotal role in enhancing the flapping motion of foils [3,12]. Lauder et al. [56] strengthened the leading edge of fish pectoral fins that enhanced propulsion efficiency compared to fins with uniform stiffness. Tapering allows greater trailing edge displacement across various actuation frequencies [57]. Zhu et al. [58] numerically examined the flow past a circular cylinder with evenly distributed rectangular ribs at a low Reynolds number to study the wake characteristics and their effect at different positions of the cylinder. Sirinavasa et al. [59] used multiple fins to see how the variation in stiffness influences the propulsion performance. Fish achieves high propulsion through complex fin–body interactions where leading edge vortices from the caudal fin contribute significantly to generating most of the thrust [60]. Zhu et al. [61] explored how fin-shaped strips affect flow dynamics, vibration amplitude, and energy harvesting efficiency using a fluid–structure interaction, and the influence of strip size and orientation was analyzed for designing more efficient hydrokinetic energy converters. Flow around a cylinder with fin-shaped strips at Re = 60–180 was aimed to enhance hydrodynamic forces and offers insights into fluid–structure interaction [62].

According to previous studies there is very limited work on the passive oscillations of foils exhibiting pure flapping or pitching motions. Pure passive pitching motion can be helpful in energy harvesting and propulsion due to its relatively simple mechanism. The strategies can be devised to improve their pitching characteristics by using appendages. This research aims to study and improve the pitching performance of the teardrop-shaped hydrofoil in the low Reynolds number range using a pair of biomimetic appendages on its sides. The symmetric foil has a round leading edge and wedge-shaped trailing edge with pitching center at the center of the round leading edge. Pitching characteristics, i.e., pitching amplitude, and frequency, etc., are vital in enhancing the foil pitching in terms of energy extraction and propulsive performance for underwater applications. The focus of the current work is to increase the pitching amplitude and frequency of pure passive pitching hydrofoil.

## 2. Experimental Setup

### 2.1. Experimental Model

**Figure 1:**
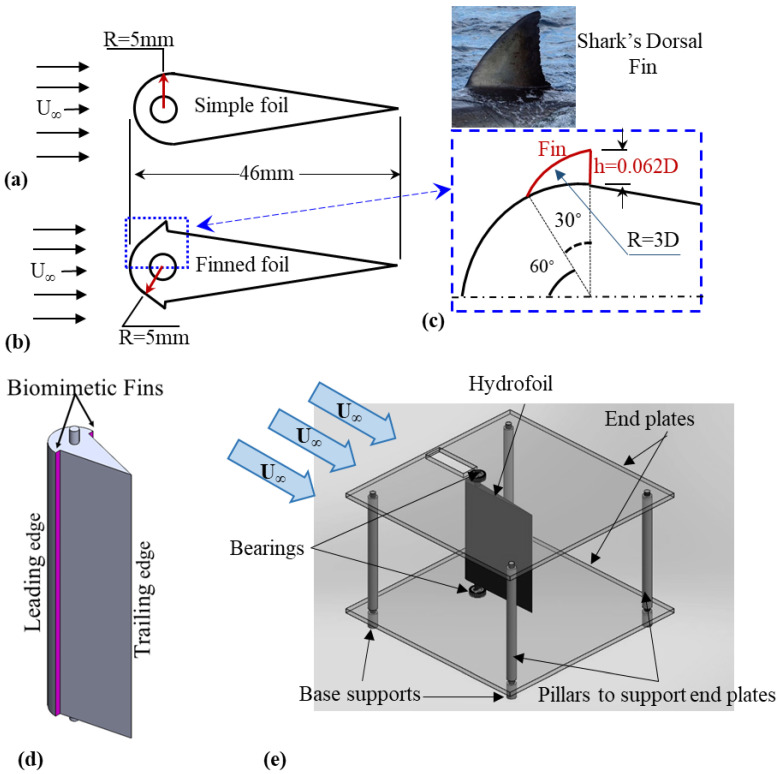
Schematic representations of (**a**) a simple hydrofoil, (**b**) a finned hydrofoil, (**c**) cross-sectional view of fin strips, (**d**) isometric view of finned foil, (**e**) illustration of the hydrofoil mounting configuration within the experimental setup, featuring end plates and pillar supports.

The experiments were performed in water channel using a teardrop shape hydrofoil (referred to as “foil” for the rest of this article) made of Aluminum (ρ = 2700 kg/m^3^) as shown in Figure 1a. The foil has a round leading edge of radius (R) with straight sides downstream of the maximum thickness point extended till trailing edge. The maximum thickness is at 0.11c from the leading edge, where c is chord length of foil. The center of the leading edge is also the maximum thickness point of the foil and is the pitching center as well, and chord length of the foil is c = 0.046 m (Figure 1a). To improve the pitching characteristics, two biomimetic fin-shaped strips [62] inspired by the shark dorsal fin were placed symmetrically on the leading edges of the foil as shown in Figure 1b. The fin strips have arc-shaped leading surfaces oriented toward the incoming flow and flat trailing faces that sit perpendicular to the foil surface. The rigid dorsal fins can modify the flow through vortex shedding, enhancing thrust and control. They showed direct contribution to propulsion through thrust augmentation in species such as sharks and tuna [63]. The fin geometry and its placement position on the leading edge are shown in Figure 1c,d.

During the water channel testing, the foils were placed between the two end plates with dimensions of 0.4 m × 0.3 m (L × W), each made of 0.006 m thick plexiglass. Pair of single-row deep groove ball bearings (626z) (known for low starting torque and minimal frictional resistance) with bore diameter of 0.006 m, outer diameter of 0.019 m, and width of 0.006 m was tightly fit in the end plates, 0.15 m from the plate’s leading edge. To quantify the bearing friction, an assessment of the rotational bearing friction and an assessment of the rotational friction torque were conducted and used to estimate the non-dimensional friction coefficient, as outlined below:

- Measured Friction Torque (Tₓ) = 2.3 × 10^−5^ Nm.
- Characteristic Torque (T_0_) = 6.0 × 10^−3^ Nm.
- Non-dimensional Friction Coefficient (Cₓ) = 0.0038 (at maximum Reynolds number).

This shows that the bearing torque is sufficiently low so as not to dominate the restoring force from fluid–structure interaction, thus allowing the pitching motion to remain passively driven by the flow-induced motion [64]. The foil is installed between two end plates with bearings at 90° from linear axis and is running clearance (RC) fit according to ANSI B4.1. The bearings restrict any heaving motion of foil and allow pure passive pitching motion only. The leading edges of the end plates were tapered to ensure a uniform flow at the entrance of experimental setup. Both end plates were parted away by 0.2 m using four rods placed at four corners with dimensions of 0.006 m × 0.2 m (D × H), each (Figure 1e).

The experimental setup was positioned in water channel so as to keep the lower end plate 0.1 m above the floor of water channel using four circular base supports, each measured 0.02 m × 0.1 m (D × H). The lifting of the lower end plate above the channel surface prevents the shear effects/boundary layer of the channel bed from interacting with the foil and ensures uniform free-stream flow to interact with the foil. The upper end plate remains 0.1 m lower than the free surface, which effectively damps the free-surface effects and influence on the pitching motion.

### 2.2. Water Channel

**Figure 2:**
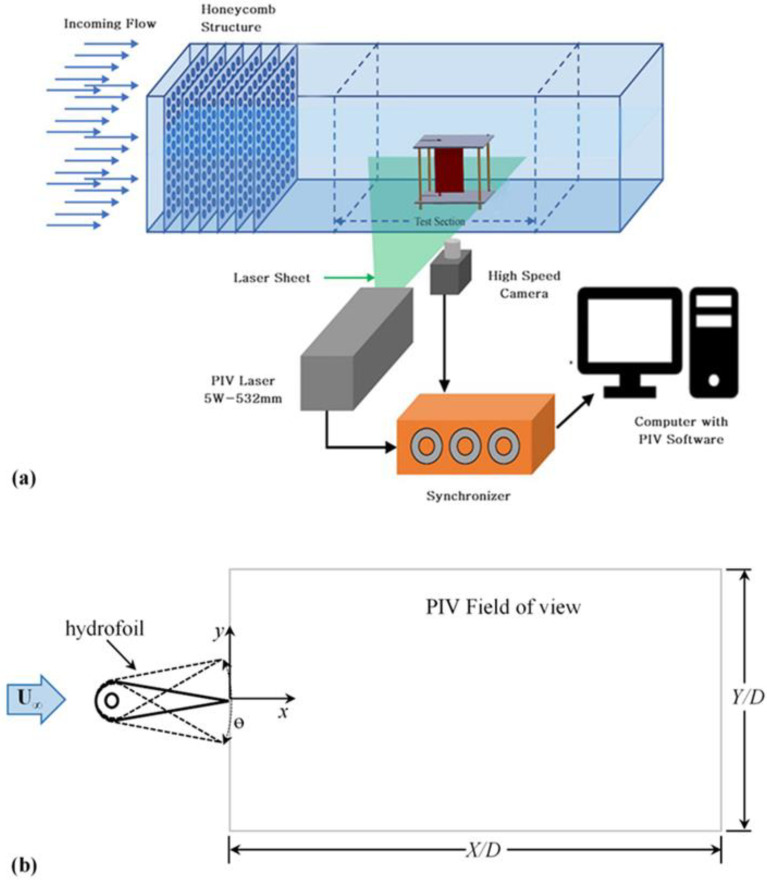
(**a**) Experimental setup for PIV and videography measurements. (**b**) Schematic representation of the free-pitching foil and the corresponding PIV field of view. The solid line indicates the mean position of the foil, while the dashed lines represent its extreme oscillatory positions.

Experiments were conducted in a low-speed, closed-loop water channel as shown in Figure 2a, having test section of 2 × 0.4 × 0.5 (L × W × H) in meters (m). The test section was constructed from glass, making it transparent and clear to observe the foil’s motion. The centrifugal pump was driven with a 10 HP motor, and its RPM was controlled by Delta electronics low-horsepower VFD (Variable Frequency Drive) operating between 1 and 50 Hz.

The free-stream velocity (U∞) of the water channel can be varied from 0.05 m/s to 0.3 m/s with variation in flow velocity at 0.006 m/s per Hz of VFD at ambient conditions. The water was introduced into the channel sump via a pump after undergoing treatment through a Reverse Osmosis (RO) water purification system. This process effectively removes suspended particulates, sediments, and impurities, ensuring the delivery of clean water at ambient conditions. This is particularly important for the PIV measurements. Four Aluminum honeycomb structures (1.83 m × 0.5 m × 0.254 m, W × H × T) with hexagonal openings were positioned 0.6 m apart, each followed by four mesh layers (wire thickness: 0.0001 m; mesh size: 0.001 m × 0.001 m, L × W). This arrangement stabilizes the flow and reduces turbulence before the test section by breaking the large eddies introduced due to the pumping of the flow. The large swirl of the flow and eddies introduced were minimized, thus creating a uniform flow profile for precise measurements. The turbulence intensity in the test section is less than 2%. This ensures high-quality flow measurements and enhances the precision of experimental results and data collection.

### 2.3. Videography

To visualize the pitching characteristics, the videos were recorded by a high-speed camera “Sony Cyber-shot DSC-RX100 IV” at 50 fps (frames per second). Obtained images were processed using image processing techniques to capture pitching traces, pitching amplitudes (θ°) of trailing edge of foil, and normalized frequency response over time for all cases. An image processing MATLAB^®^ code was developed to analyze the recorded videos and to calculate the pitching amplitude and frequency. The method involves a tail point technique to track the trace of the trailing edge of the foil. By focusing on the foil’s endpoint in each processed image frame, we accurately captured and analyzed the foil’s motion dynamics and movement patterns with instantaneous data, to find flapping motion over time [65]. For each case, the video was recorded for 120 s (sec) at fixed incoming flow velocity controlled using VFD. Data was collected thrice for each velocity with an interval of 300 s for every consecutive data set. The mean values of the results obtained were based on results from three repeated runs and were finalized as per standard procedures with a precision level of more than 0.99. The interval between two readings taken for two different flow velocities was 600 s to develop flow properly before taking data.

### 2.4. Particle Image Velocimetry

The wake of the foil was investigated using a Particle Image Velocimetry (PIV) system to achieve high-resolution measurements of the velocity field. A 0.001 m thick laser sheet, produced by a 5W diode-pumped solid-state (DPSS) laser, was aligned along the foil’s mid-span, capturing a cross-sectional view of the wake. The laser operates at 10 frames per second and was synchronized with 2 Mega pixels, Imperx CLB-B1320M CCD camera. The camera’s frame rate was synchronized with the laser, thereby enhancing the temporal fidelity of the captured images. The water was seeded with hollow glass spheres (density 1.05 g/cm^3^) having average size of 10 μm to trace the flow due to their reflective properties under the laser sheet [66]. These images were processed using MICROVEC PIV^®^ software (2D2C PIV/PTV) for cross-correlation analysis to determine particle displacements across a pair of frames. This process yielded vector fields representing the velocity distribution in the wake, offering valuable insights into the flow structures and enhancing understanding of the fluid dynamics surrounding the foil [64,65]. For each case, 1000 image pairs were captured and processed to obtain the averaged wake behavior in terms of time average and fluctuating velocity components. The PIV results were taken for 0 ≤ X/D ≤ 13 along the streamwise direction and −3.5 ≤ Y/D ≤ +3.5 in transverse direction. The X/D = 0 and Y/D = 0 are the locations of the trailing edge of the foil in non-pitching condition in the streamwise and transverse direction (Figure 2b).

### 2.5. Validation of Experimental Setup

**Figure 3:**
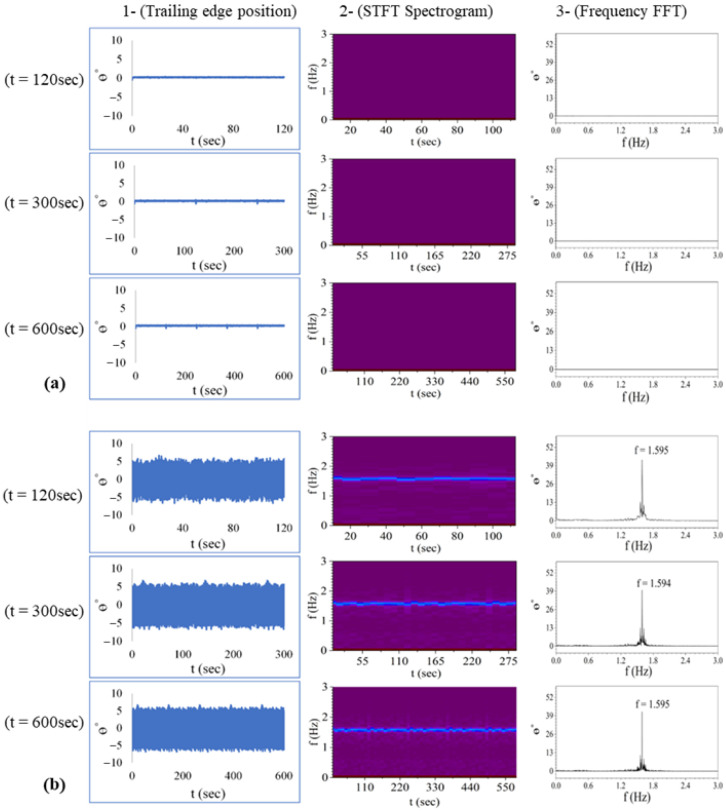
Validation of experimental setup (**a**,**b**) shows the results at Re = 1300 and 3200, respectively, with column 1 showing the hydrofoil’s trailing edge position, column 2 and 3 illustrating frequency using STFT spectrogram and FFT, respectively, for different time steps and Reynolds numbers.

To check the repeatability and reproducibility of the obtained results, the plain foil was tested for Re = 1300 (No Pitching) and Re = 3200 (pitching with maximum amplitude). The Reynolds number, Re = ρ U_∞_D/µ, where ρ is density of water, U_∞_ is free-stream velocity, D is the maximum thickness of foil, and µ is dynamic viscosity of water. Measurements (videography) were carried out for time intervals of 120 s, 300 s, and 600 s to analyze the impact of data set size on the results or intermittency of the pitching behavior. To establish this, the setup was placed in the channel with a flow at a certain fixed velocity for 600 s, which is adequate enough to avoid any transients in the flow. Data was recorded for 600 s first, followed by a recording interval of 300 s and 120 s, with a pause of 300 s between each recording. At each Re, the trailing edge position (θ°) of the foil was plotted against time (sec) to detect any variations in amplitude with time. The Short-Time Fourier Transform (STFT) was applied to display resultant spectrogram of time-varying frequency information for different data sets. Fast Fourier Transform (FFT) was performed to obtain the dominant frequency to compare the results obtained with different datasets. For three data sets obtained at Re = 1300 and Re = 3200, the results of pitching amplitudes (θ°), the spectrogram, and dominant frequencies are plotted in Figure 3a and Figure 3b, respectively. Minor differences in average frequencies and amplitudes demonstrated the reliability and consistency of the experimental setup, process, and obtained results. The consistent pattern of increasing amplitude and frequency with Reynolds numbers underscores the system’s sensitivity to changes in flow conditions and validates its effectiveness in capturing the dynamics of pitching behavior.

To evaluate the presence of hysteresis effects, measurements were conducted by varying the tunnel velocity for simple foil in two sequential steps: initially increasing from Re 1300 to 3200, followed by a reverse sequence from 3200 back to 1300, without stopping the flow. The frequencies obtained in either ramp-up or ramp-down situations demonstrated negligible differences. This consistency confirms the absence of any significant hysteresis effects and validates further the reliability and repeatability of the experimental setup and results.

## 3. Results and Discussions

### 3.1. Pitching Amplitude

**Figure 4:**
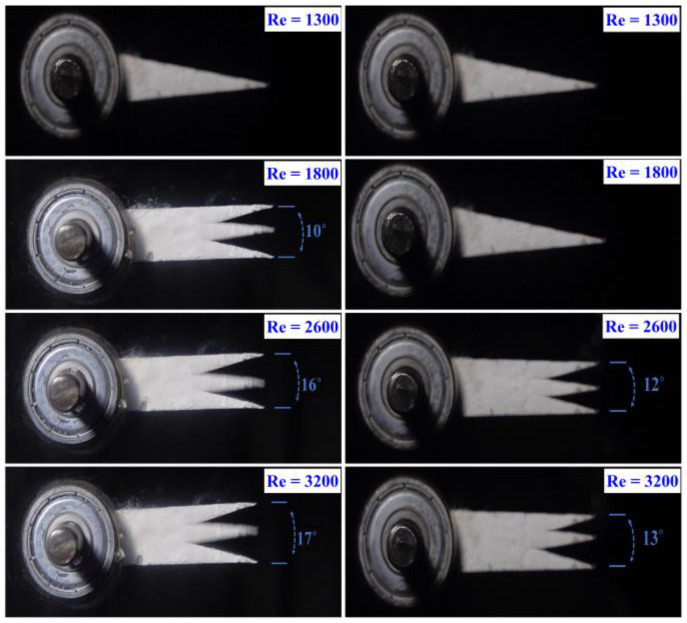
Flapping trace of finned (**Left**) and simple (**Right**) foil at different Reynolds numbers.

**Figure 5:**
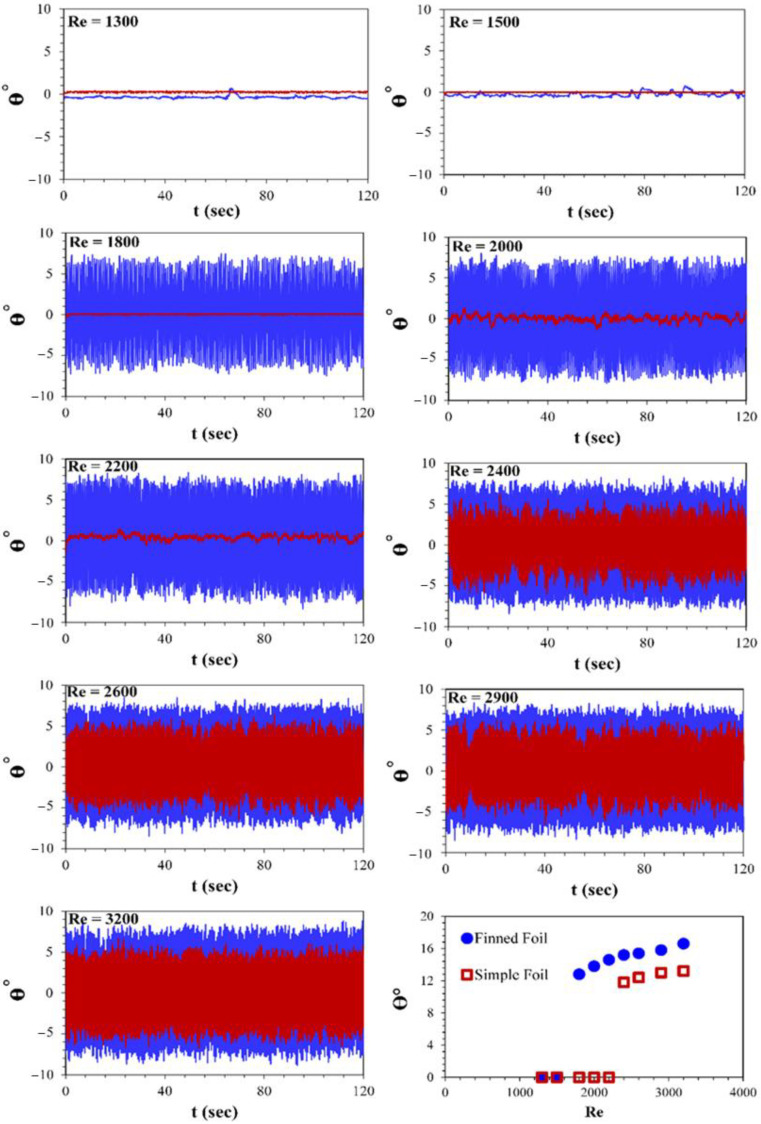
Time-history amplitude response of Finned (Blue) and Simple (Red) foils at different Reynolds numbers.

Images of foils were extracted from video recordings using StarStaX software to analyze their pitching motion for the four selected cases, which will be discussed in the following sections. The software was employed to capture frames at extreme positions of the oscillation cycle, providing a clear trace of the foil’s motion. These traces effectively illustrate the dynamic behavior and kinematic patterns of the foils during their pitching movement. The figure demonstrates the motion of a finned foil on the left and a simple foil on the right, highlighting the differences in their trajectories and performance characteristics as shown in Figure 4. The analysis of pitching amplitude responses for the two foils, the Simple Foil (red line) and the Finned Foil (blue line), as depicted in Figure 5. Figure 5 presents a 120 s time series of pitching amplitudes for both foils, showing average and peak pitching amplitudes as functions of Reynolds number. At low Reynolds numbers (Re = 1300 and 1500), both foils exhibit negligible oscillations, as the weak vortex-shedding forces are insufficient to generate significant pitching motion. However, as the Reynolds number increases to 1800, the finned foil begins to exhibit pitching, with an average amplitude of approximately ±5° about the horizontal axis during crest and trough movements in a sinusoidal pattern. The peak pitching amplitude for the finned foil reaches ±6.4° at this Re, while the simple foil remains nearly static, producing no significant oscillation or useful output. The fins at the leading edge of the finned foil generate localized flow disturbances and promote earlier separation. This mechanism enhances wake turbulence and increases the frequency and strength of vortex shedding, driving higher pitching amplitudes.

As the Reynolds number further increases to 2000 and 2200, the finned foil’s average pitching amplitude rises to ±5.2° and ±5.5°, respectively, with peak values of ±6.9° and ±7.2°. The simple foil, however, remains largely static at these Reynolds numbers. The fins’ sharp features facilitate the generation of strong leading-edge vortices (LEVs) [62], which destabilize downstream flow and amplify trailing-edge vortex shedding. These effects result in intensified vortex-induced vibrations and more complex wake structures. At Re = 2400, the simple foil begins to exhibit pitching as viscous forces are overcome. Despite this, the difference in performance remains evident, with the finned foil achieving average and peak pitching amplitudes of approximately ±5.6° and ±7.6°, compared to ±3° and ±5.9° for the simple foil. The fins on the finned foil increase the wake’s effective width by introducing additional separation points. The interaction between vortices shed from the fins and those formed at the trailing edge contributes to a broader, more turbulent wake and higher pitching amplitudes. As the Reynolds number increases to 2600 and 2900, the finned foil’s average pitching amplitudes reach ±6° and ±6.1°, with peak values of ±7.7° and ±7.9°, respectively. Meanwhile, the Simple Foil achieves average amplitudes of ±3.4° and ±4°, and peak values of ±6.2° and ±6.5°. The fins amplify vortex-induced vibrations, increasing the magnitude of unsteady forces acting on the foil. The stronger vortices generated by the fins transfer more energy from the flow to the foil, resulting in greater pitching amplitudes.

At the maximum Reynolds number studied (Re = 3200), significant differences persist between the foils. The finned foil achieves average and peak pitching amplitudes of ±6.7° and ±8.3°, respectively, compared to ±4.8° and ±6.6° for the simple foil. At higher Reynolds numbers, intensified turbulence and stronger interactions between the boundary layers enhance wake dynamics. The finned foil, with its larger leading edges, generates stronger LEVs that exacerbate separation dynamics, destabilizing the flow [2,62] and further amplifying pitching behavior. Table 1 presents the percentage increase in pitching amplitude of the finned foil relative to the simple foil. In the initial phase, both foils remain stationary, exhibiting no motion. During the second phase, the finned foil begins pitching, demonstrating a 100% increase in amplitude compared to the static simple foil; the term 100% is used because of no pitching motion for the simple foil at those certain Reynolds numbers. As the third phase commences, with the simple foil initiating its pitching motion, the average increase in pitching amplitude of the finned foil is observed to be 25%, indicating its superior performance characteristics. The percentage differences in both amplitude and frequency are calculated using the simple foil as the reference. The expression used for calculating the pitching amplitude of foil is the following:

(1) $$\;\%\;Difference\;in\;\theta {}^{\circ}=\left(\frac{\theta {}^{\circ}Finned\;Foil\;\;-\theta {}^{\circ}Simple\;Foil}{\theta {}^{\circ}Simple\;Foil}\right)\times 100$$

### 3.2. Pitching Frequency

**Figure 6:**
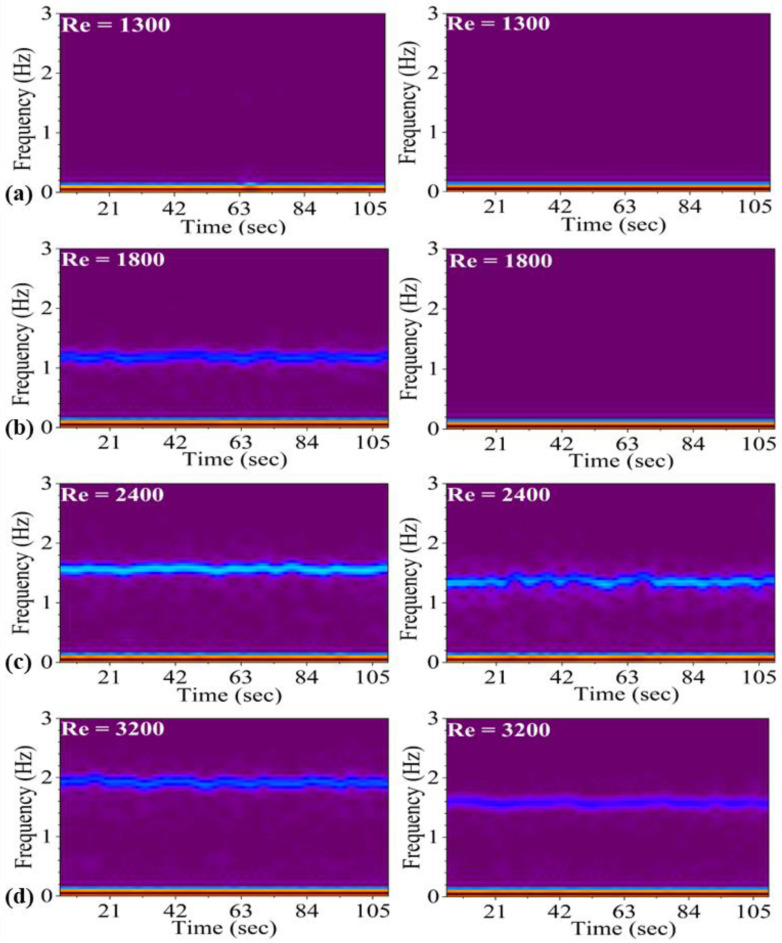
Frequency response of finned and simple foils, (**a**–**d**) in spectrogram form with respect to time.

Figure 6a–d present the time-varying frequency distribution of Finned and Simple Foils across different Reynolds numbers, derived from the spectrogram of the Short-Time Fourier Transform (STFT) analysis. Each graph demonstrates the temporal evolution of dominant frequencies [67], revealing how specific frequency components vary with increasing Reynolds numbers and reflecting changes in the fluid dynamics around the foils. This comparative analysis offers insights into how the two foil designs interact with the flow, influencing their pitching performance under varying Reynolds numbers. The frequencies of the pitching foils were measured for Reynolds numbers ranging from 1300 to 3200 over a 120 s time period in each case.

The STFT spectrograms for finned and simple foils are shown in Figure 6a–d, representing time-frequency analysis at different Reynolds numbers in each case. Both finned and simple foils remain static at Reynolds numbers of 1300 and 1500, indicating no pitching motion. The time-frequency variation (TFV) images in Figure 6a show no frequency signal throughout the 120 s data, suggesting the absence of vortex shedding or oscillatory motion at Re = 1300, due to weak leading-edge vortices, weak hydrodynamic forces, and a larger mass of foil.

At Re = 1800, the finned foil begins to pitch (Figure 6b), where a stable line at a pitching frequency of 1.22 Hz is observed throughout the 120 s interval. In contrast, the simple foil remains static with no pitching motion. This disparity is attributed to the geometric differences between foils. The vortex shedding from the leading edges of the fins separates the flow from the foil surface and generates stronger vortices compared to the simple foil. These larger vortices result in increased hydrodynamic forces and strong separated flow, which enhance the pitching motion. The suction effect at the opposite end of the foil is also amplified due to the larger vortices, leading to greater pitching behavior in the finned foil that initiates pitching motion. As the Reynolds number increases to 2000, the pitching frequency of the finned foil rises to 1.31 Hz, while the simple foil remains static. At a Reynolds number of 2200, the frequency approaches 1.47 Hz for the finned foil, with the simple foil still showing no pitching motion. At Reynolds number 2400, both foils are pitching, with the finned foil exhibiting a frequency of 1.62 Hz and the simple foil showing a lower frequency of 1.36 Hz (Figure 6c). The pitching frequencies of both foils continue to increase with further increases in Reynolds numbers. At Reynolds number 2600, the pitching frequencies for the finned and simple foils reach 1.69 Hz and 1.37 Hz, respectively. The monotonic increase in pitching frequency persists with rising Reynolds numbers. At Reynolds number 2900, the value of frequency reaches 1.84 Hz for the finned foil and 1.50 Hz for the simple foil. At Reynolds number 3200, the TFV images showing frequencies reach their maximum value of 1.93 Hz and 1.60 Hz for the finned and simple foils, respectively (Figure 6d). This value represents the upper limit of this study due to experimental constraints. Despite being in the lower transitional flow regime, the higher water speeds produce significant changes, such as earlier phase reversal and faster stroke transitions, indicative of increased pitching velocities.

**Figure 7:**
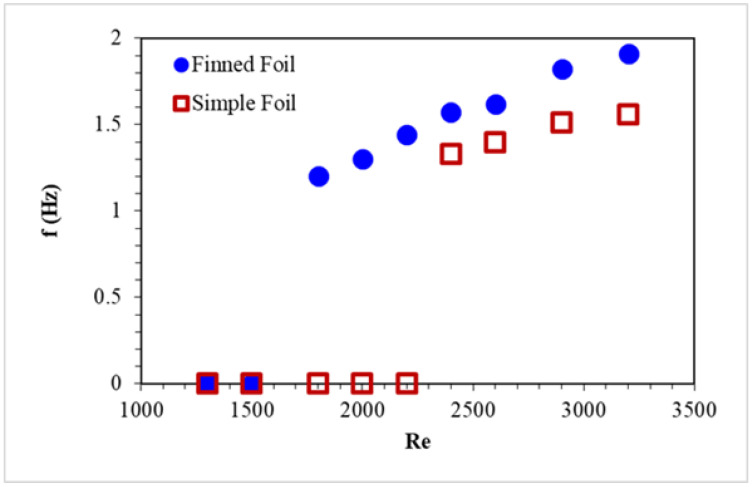
Maximum frequency variation in finned and simple foil in absolute values.

Figure 7 summarizes the frequency response of both foils across varying Reynolds numbers, highlighting the steady increase in pitching frequency with increasing Reynolds number. At very low Reynolds numbers, both foils display no pitching motion insufficient to generate measurable frequencies. Notably, the finned foil begins pitching at lower Reynolds numbers while the simple foil remains static. The increased frequency observed in the finned foil at low water speeds is attributed to the larger vortex shedding area created by the fins at the leading edge. This enhancement also increases the wake region behind the trailing edge. As Reynolds numbers exceed 2300, the simple foil begins pitching, showing flapping characteristics. However, the finned foil consistently exhibits higher pitching frequencies than the simple foil across all cases. From Table 2, it is evident that, at the maximum Reynolds number, the finned foil shows an overall 21% higher pitching frequency, and, at the lowest Reynolds number, finned foil shows 100% higher pitching frequency than the simple foil, as it shows no pitching motion at all. This increase directly enhances the energy-harvesting potential of the finned foil. The stable frequency lines over the extended period indicate that each foil maintains a steady vortex shedding frequency, corresponding to a periodic flow pattern in the wake region. The periodic vortex shedding ensures consistent oscillations of the foils. Such behavior is critical for applications requiring periodic aerodynamic forces, i.e., autonomous propulsion in underwater vehicles and energy harvesting [2].

The equation to calculate the percentage difference in frequency is as follows:

(2) $$\;\%\;Difference\;in\;f=\left(\frac{f\;Finned\;Foil-f\;Simple\;Foil}{f\;Simple\;Foil}\right)\times 100$$

### 3.3. Mean Streamwise Velocity

**Figure 8:**
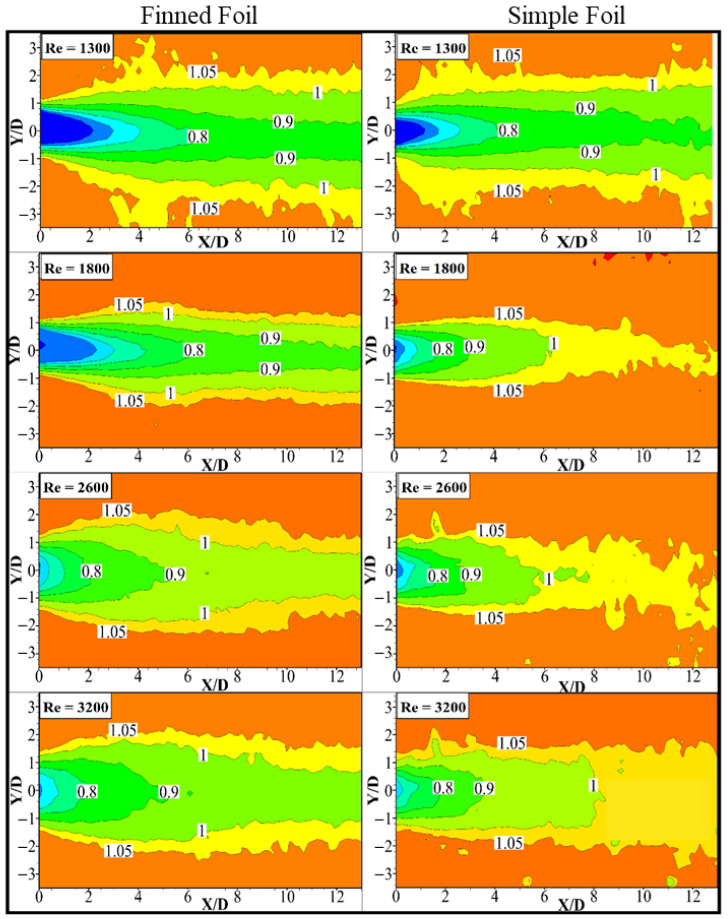
Contours of normalized mean streamwise velocity fields of pitching foils at different Reynolds numbers.

Particle Image Velocimetry (PIV) measurements were conducted to investigate the downstream wake characteristics of a foil. The non-dimensionalized time-averaged streamwise velocity ($\bar{u}*$ = $\;u/U_{\infty }$) fields are presented in Figure 8, with contours of finned foil shown on the left and simple foil on the right. Both axes of the contours are non-dimensionalized with the foil diameter. A uniform contour increment of $∆\bar{u}*$ = 0.1 is applied to facilitate easier comparison, with $\bar{u}*=\;$1.05 taken as the maximum contour level. This ensures a consistent basis for comparing the influence of wake regions generated by the pitching foils. Three comparative situations based on foil pitching are selected at different Reynolds numbers (Re) as mentioned in the previous sections and evaluated for wake. At Re = 1300, where both foils remain static and do not exhibit any pitching motion, the wake is wider with a slower wake recovery. The contour level $\bar{u}*$ = 0.9 approaching the wake center line (Y/D = 0) is considered to compare the wake recovery. At Re = 1300, $\bar{u}*$ = 1 for both foils does not approach Y/D = 0 in the flow field region observed here X/D ≤ 13. As the Reynolds number increases to Re = 1800, the wake recovery accelerates with $\bar{u}*$ = 0.9, approaching the wake center line at X/D = 3, prominently for simple foil due to the increase in flow velocity and narrower wake. Finned foil on the other hand has very slow recovery of the wake that is due to the early pitching motion of the foil at this Reynolds number compared to the simple foil. Both foils observed pitching at Re = 2600 with different pitching frequencies and amplitudes, as observed earlier in the previous section. The wake recovery for the simple foil is slightly faster than at Re = 1800 for the finned foil, the wake $\bar{u}*$ = 0.9 approaches the wake center line at X/D = 5.5. This is due to the higher pitching amplitude of the finned foil compared to that of the simple foil. The trend of wake recovery for finned and simple foil remains the same as Re increases to 3200, faster for the simple foil and slower for the finned foil. In this study, a parameter called the maximum influenced wake width [68], denoted as *w*′, is defined by measuring the maximum width of a specific contour level at a predetermined downstream location relative to the pitching foil. Specifically, here *w*′ represents the transverse extent of the mean streamwise velocity $\bar{u}*$ = 1 above and below the wake symmetric line. This measurement is taken at a location where the maximum width of the wake downstream of the trailing edge of the foil for each case is observed. At Re = 1300, the simple foil has a maximum influence wake width of *w*′ = ±1.44 at X/D = 5, and, correspondingly, the finned foil has *w*′ = ±1.65 at X/D = 6.1.

The wider wake-influenced region for finned foil is possibly due to stronger separation, which results in a larger and wider wake region when both foils are non-pitching. The influenced wake region remains fixed downstream of X/D = 5 and X/D = 6.1 for simple and finned foils. For the scenario when the finned foil starts pitching while the simple foil is still static (Re = 1800), *w*′ = ±1.04 is observed at X/D = 2.73 for simple foil, whereas *w*′ = ±1.43 is observed at X/D = 4.65 for the finned foil. The reduction in *w*′ and upstream movement of its location of occurrence for both foils is due to the shear thinning associated with the increase in Reynolds number. A comparatively large reduction in *w*′ for the simple foil is linked with its non-pitching behavior. A slight narrowing of the wake is observed for the finned foil due to the entrainment of the high-speed flow into the wake. As both foils pitch when the Reynolds number approaches Re = 2600, *w*′ = ±1.16 and *w*′ = ±1.7 are observed, respectively, for simple and finned foils. This increase in *w*′ for both foils is associated with the large amplitude pitching observed for both cases. The location of occurrence, on the other hand, is observed to be continually moving upstream towards the trailing edge of the foil. Downstream of the location (X/D) where *w*′ is observed, the wake starts to narrow down sharply for the finned foil. This is due to large amplitude oscillations, which possibly result in a turbulent wake and faster wake recovery. At Re = 3200, the larger influenced wake widths of *w*′ = ±1.35 and *w*′ = ±1.8 are observed for simple and finned foils, respectively. Now, the streamwise location (X/D) of maximum *w*′ started shifting downstream, opposite its behavior at lower Reynolds numbers. Maximum *w*′ is observed at X/D = 2.9 and 3.45 for simple and finned foils, respectively.

The drag force and corresponding drag coefficients in this study were estimated based on the momentum–balance approach [41], due to similarity in the shape of base-line foil. The current experimental setup provided a limited flow field of view, specifically constrained to 0 ≤ X/D ≤ 13 in the streamwise and −3.5 ≤ Y/D ≤ +3.5 in the transverse direction. This limitation, primarily due to available equipment and facility constraints, restricted the full spatial resolution required for highly accurate drag computation. As a result, drag estimations for a complete wake profile in the transverse direction were not feasible, and certain parameters critical for comprehensive drag analysis could not be captured.

**Figure 9:**
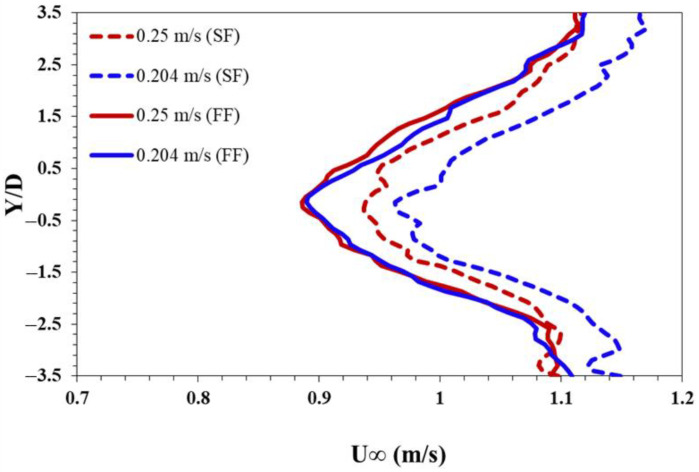
Velocity profile of wake at different velocities.

Nevertheless, for a subset of velocities of two cases aligned with those analyzed for PIV velocity contours Figure 9, the ratios of the drag coefficients for the finned foil relative to the simple foil were calculated at different flow velocities. Due to the lack of pitching motion at lower flow velocities for either simple or finned foil, only the higher velocity cases were considered relevant, yielding drag coefficient ratios of 0.665 and 0.851 at 0.25 m/s and 0.204 m/s, respectively.

### 3.4. Root Mean Square (RMS) Streamwise Velocity

**Figure 10:**
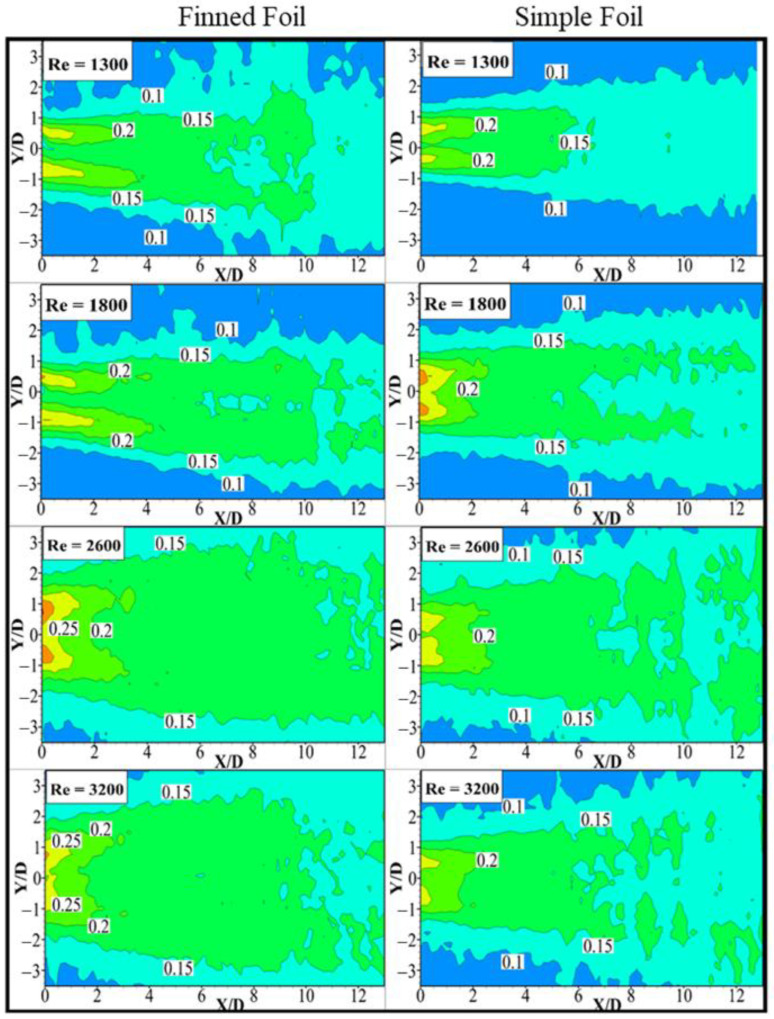
Contours of normalized RMS streamwise velocity fields of pitching foils at different Reynolds numbers.

The contours of root-mean-square values of time-averaged streamwise velocity (${\bar{u}}_{rms}^{*})$ fields for the specified phases are illustrated in Figure 10. A uniform contour increment of ${\bar{u}}_{rms}^{*}$ = 0.05 is applied to all ${\bar{u}}_{rms}^{*}\;$ values to facilitate easier comparison. The influenced wake width (*w*′) utilized for comparing the regions of influence of the pitching foil in this study is defined based on the transverse positions corresponding to the contour level of ${\bar{u}}_{rms}^{*}$ = 0.15 at a fixed streamwise distance X/D = 4 downstream from the centerline of wake symmetry for uniform comparison. The smallest influenced wake width *w*′ = 2.92 and 2.44, while the widest influenced wake width *w*′ = 4.94 and 3.59 for finned and simple foil is observed, in the first and third phases, respectively, at X/D = 4. In the second phase, it is observed that the influenced wake width (*w*′) for the finned foil exhibits a value similar to that in the first phase, despite the higher Reynolds number in the second phase. This behavior is attributed to the initiation of the foil’s pitching motion, which influences the wake structure. At lower pitching values, the generated vortices tend to be weaker and more compact, leading to a narrower wake width [46]. It is observed that the influenced wake width increases with rising Reynolds numbers, reaching maximum width because of increasing oscillation amplitudes.

However, at the highest Reynolds number examined in this study, the wake width slightly decreases. This reduction is attributed to the rise in shedding frequency, which results in the formation of more compact vortices near the foil. Further downstream, the wake undergoes broadening again due to vortex merging effects [69]. A comparison of both foils, as shown in the figure, demonstrates that the finned foil notably outperforms the simple foil in terms of the influenced wake width. The average drag coefficient is influenced by both the formation length $L_{f}^{*}$ and the influenced wake width *(w*′), with the larger *w*′ associated with higher drag forces. Notably, the influenced wake width (*w*′) has a more pronounced impact on drag forces compared to the formation length ${\;L}_{f}^{*}$. In contrast, the wake width (*w*∗) parameter exhibits minimal influence on the average drag forces [70]. Conversely, this broader influenced wake width is crucial for enhancing energy harvesting potential, primarily due to the generation of stronger vortex formations.

### 3.5. Mean Transverse Velocity

**Figure 11:**
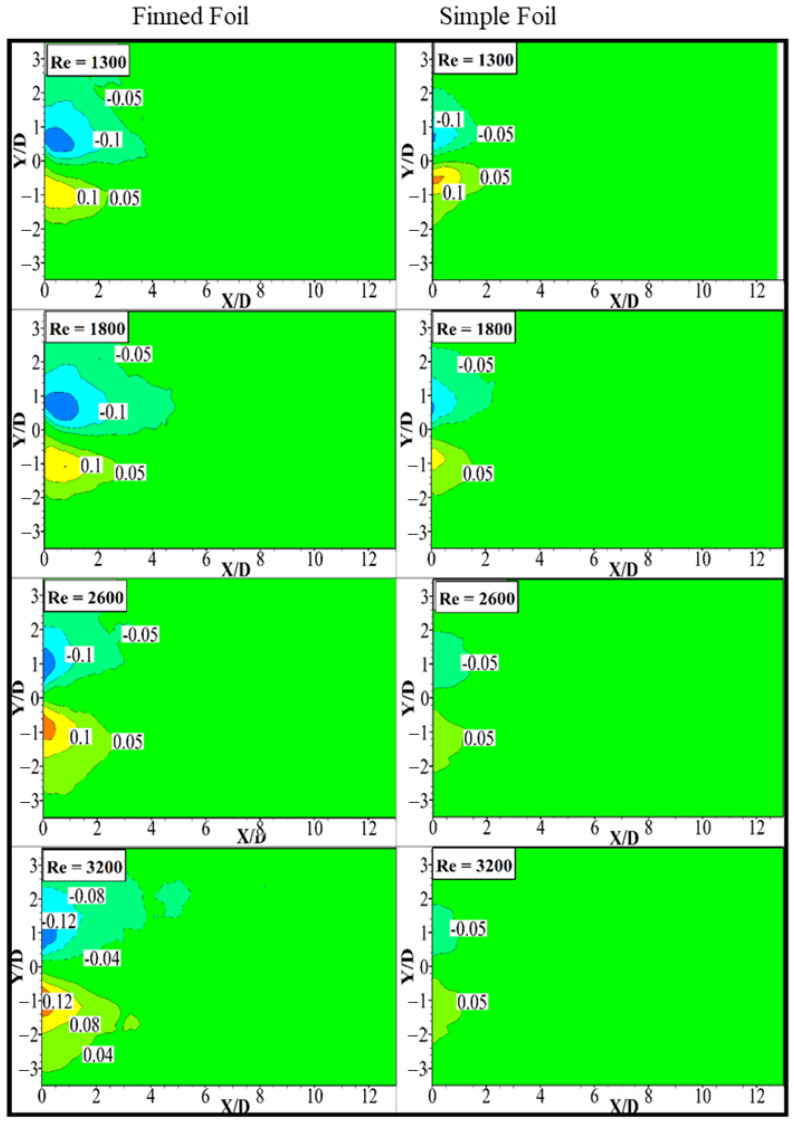
Contours of normalized mean transverse velocity fields of pitching foils at different Reynolds numbers.

Time-averaged transverse velocity contours for the identified phases are presented in the accompanying Figure 11. In these contours, positive transverse velocity values are depicted using solid lines, whereas negative transverse velocity values are illustrated with dashed lines. For all the examined cases, it is observed that, on the upstream side of the foil, the flow acquires a transverse velocity component (ῡ*=v/U∞) as it adjusts to navigate around the foil. Subsequently, this transverse velocity reverses direction as the flow crosses the foil towards the downstream side, eventually dissipating at a certain distance downstream from the foil’s center. On the downstream side, the transverse velocity component demonstrates a distinct pattern; i.e., it is negative for Y/D > 0 and positive for Y/D < 0. This behavior is attributed to the entrainment of ambient fluid into the wake. The maximum magnitude of the transverse velocity observed in each scenario serves as an indicator of entrainment affinity. A higher maximum transverse velocity correlates with stronger entrainment. As the flow approaches the wake symmetry line at Y/D = 0, a horizontal axis of symmetry passing through the foil’s center; it decelerates and aligns parallel to the incident flow. Subsequently, the flow accelerates above and below the symmetry line [69].

In all the selected cases, the downstream flow patterns exhibit similar characteristics. However, variations in the strength of the maximum lateral velocity are evident across different phases. The contours comparing the transverse velocity (ῡ*) for the finned foil (on the left) and the simple foil (on the right) highlight that the finned foil generates a stronger lateral velocity component. This effect is attributed to pronounced flow separation and larger vortex generation caused by the presence of fins. The greater magnitude of the transverse velocity contours with greater pitching amplitude observed for the finned foil indicates faster entrainment compared to the simple foil. This accelerated entrainment enhances mixing and improves wake recovery. Furthermore, the faster entrainment promoted by the finned foil contributes to higher energy harvesting potential by sustaining stronger oscillations and facilitating efficient energy transfer. The findings emphasize that geometric modifications, such as the inclusion of fins, play a crucial role in optimizing fluid dynamics and improving the overall performance of energy harvesting systems.

### 3.6. Root Mean Square (RMS) Transverse Velocity

**Figure 12:**
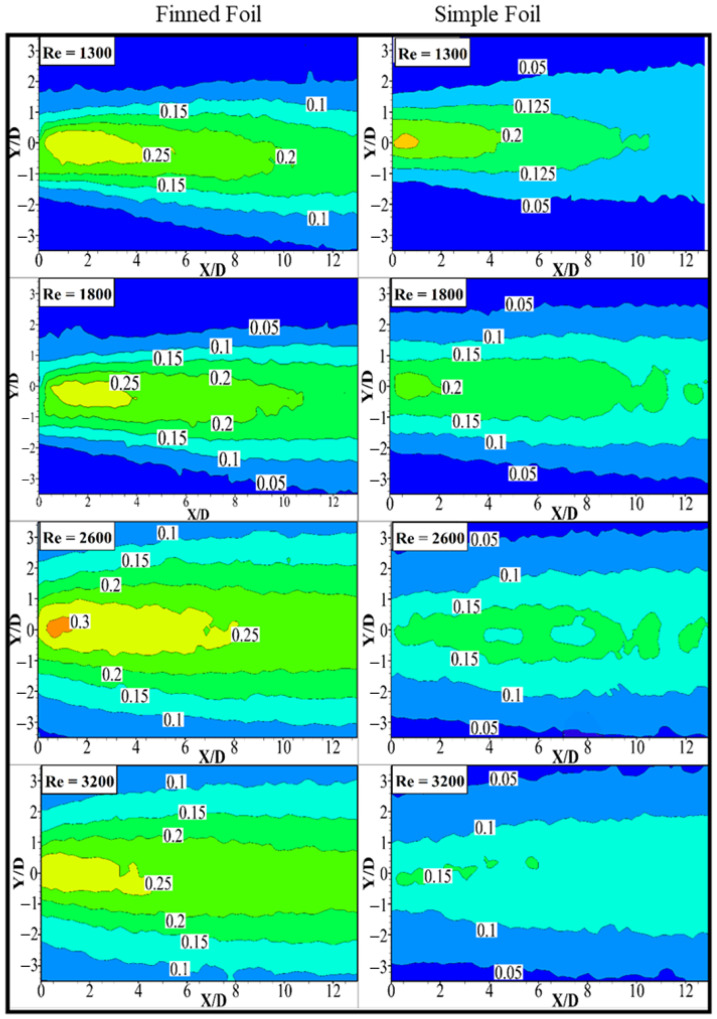
Contours of normalized RMS transverse velocity fields of pitching foils at different Reynolds numbers.

Unlike the ${\bar{u}}_{rms}^{*}$ contours, the ${ῡ}_{rms}^{*}$ contours exhibit a distinct peak along the wake centerline behind the downstream cylinder for all the investigated cases. As evident from Figure 12, the location of the maximum ${ῡ}_{rms}^{*}$ within the wake region for the finned foil (left) is consistently higher than that observed for the simple foil (right) across all configurations presented in Figure 12. This difference primarily arises due to the influence of the fin in the static case, which alters the near-wake vortex dynamics by modifying the shedding characteristics.

In the pitching scenarios, the effect becomes more pronounced due to the combined influence of increased oscillation amplitude and shedding frequency. Specifically, larger oscillation amplitudes amplify the vortex-induced vibrations, leading to stronger transverse flow oscillations, while the enhanced shedding frequency introduces higher wake turbulence intensities. These effects collectively contribute to faster flow entrainment in the wake region of the finned foil, as discussed in prior sections. The fin structure likely increases the effective cross-sectional interaction with the flow, generating stronger spanwise vorticity and promoting earlier vortex roll-up. Consequently, this facilitates more efficient momentum transfer, enhancing wake recovery and intensifying turbulence levels. In contrast, the simple foil lacks these geometric enhancements, resulting in a relatively lower transverse velocity fluctuation intensity. Overall, the higher ῡ*rms levels observed for the finned foil underscore its capability to modify wake dynamics through both passive and active mechanisms. The passive contribution stems from the geometric addition of the fin, whereas the active contribution arises from the pitching motion, which reinforces vortex shedding synchronization and improves flow entrainment efficiency in the wake region.

## 4. Conclusions

This study experimentally investigates the influence of attaching a pair of biomimetic fins symmetrically on a teardrop foil with a rounded leading edge and a wedge-shaped trailing edge undergoing pure passive pitching motion at low Reynolds numbers. The experiments were conducted in a closed-loop water channel for the Reynolds number range of 1300 ≤ Re ≤ 3200, based on foil diameter. A comparative analysis between a simple teardrop foil (simple foil) and a finned teardrop foil (finned foil) was performed. Key parameters, including pitching amplitude (θ), pitching frequency (f), time average and fluctuating streamwise and transverse velocity components, were examined using high-speed videography and Particle Image Velocimetry (PIV). The principal findings are summarized as follows:

(a) Finned foil demonstrates an initiation of pitching at comparatively low Reynolds numbers than that of the simple foil. For Reynolds numbers where both foils are pitching, the finned foil exhibited a significant increase of 25% in θ compared to the simple foil. The addition of fins may result in strong flow separation; thus, pitching motion occurs at a lower Reynolds number, with a monotonic increase in pitching motion, θ, as the Reynolds number increases. The large θ of the finned foil results in a broader wake-affected region observed behind the finned foil using PIV.
(b) The “f” of the finned foil shows a 21% increase compared to the simple foil for Reynolds number where both foils undergo pitching. This enhanced pitching behavior of the finned foil thus improves the energy harvesting potential of foil due to the addition of fins.
(c) Mean and RMS streamwise velocity variations were analyzed, which shows that the presence of fins led to slower wake recovery and a wider *w*′ region, which scaled with increased pitching amplitudes. However, at higher velocities, the *w*′ of finned foil exhibited slight reductions due to a thinner shear region as the flow accelerates for high velocity and the entrainment of high-velocity fluid towards the wake center line by strong vortices.
(d) Transverse RMS velocity contours revealed that pitching foil adds strong transverse velocity components, which reverse their direction after passing the foil. The stronger lateral velocity components induced by the fins, facilitated by enhanced vortex formation, accelerated flow entrainment. This augmented entrainment promoted mixing and wake recovery, thereby increasing energy harvesting potential [2].
(e) This work focuses on enhancing the pitching characteristics of the foil using biomimetic fin strips. Such enhanced pitching behavior of the foils will be used to design and develop an active flapping-based [71] piezoelectric energy harvester [72] for offshore IoT-based applications.

## Figures and Tables

**Table 1:** The percentage increase in amplitude of the finned foil in comparison to the simple foil.

	Re	Pitching Amplitude		Percentage Increase
		Finned Foil	Simple Foil	
1	1300	0	0	0
2	1500	0	0	0
3	1800	12.8	0	100
4	2000	13.8	0	100
5	2200	14.6	0	100
6	2400	15.2	11.8	28.8
7	2600	15.4	12.4	24.2
8	2900	15.8	13	21.5
9	3200	16.6	13.2	25.8

**Table 2:** Maximum percentage frequency variation in finned and simple foil.

Sr. No.	Re	Pitching Amplitude		Percentage Increase
		Finned Foil	Simple Foil	
1	1300	0	0	0
2	1500	0	0	0
3	1800	1.22	0	100
4	2000	1.31	0	100
5	2200	1.47	0	100
6	2400	1.62	1.36	19.1
7	2600	1.69	1.37	23.4
8	2900	1.84	1.5	22.7
9	3200	1.93	1.6	20.6

## Data Availability

Provided upon request.
